# Applications of Intravital Imaging in Cancer Immunotherapy

**DOI:** 10.3390/bioengineering11030264

**Published:** 2024-03-08

**Authors:** Deqiang Deng, Tianli Hao, Lisen Lu, Muyang Yang, Zhen Zeng, Jonathan F. Lovell, Yushuai Liu, Honglin Jin

**Affiliations:** 1 College of Biomedicine and Health and College of Life Science and Technology, Huazhong Agricultural University, Wuhan 430070, China; dengdeqiang@hust.edu.cn (D.D.); lulisen@mail.hzau.edu.cn (L.L.); yangruilin2@163.com (M.Y.); 13688386186@163.com (Z.Z.); 2 Department of Biomedical Engineering, University at Buffalo, State University of New York, Buffalo, NY 14260, USA; jflovell@buffalo.edu; 3 Department of Ophthalmology, Union Hospital, Tongji Medical College, Huazhong University of Science and Technology, Wuhan 430022, China

**Keywords:** intravital imaging, cancer immunotherapy, nanoparticle, immune checkpoint inhibitor, adoptive cell therapy, immune cell tracking

## Abstract

Currently, immunotherapy is one of the most effective treatment strategies for cancer. However, the efficacy of any specific anti-tumor immunotherapy can vary based on the dynamic characteristics of immune cells, such as their rate of migration and cell-to-cell interactions. Therefore, understanding the dynamics among cells involved in the immune response can inform the optimization and improvement of existing immunotherapy strategies. In vivo imaging technologies use optical microscopy techniques to visualize the movement and behavior of cells in vivo, including cells involved in the immune response, thereby showing great potential for application in the field of cancer immunotherapy. In this review, we briefly introduce the technical aspects required for in vivo imaging, such as fluorescent protein labeling, the construction of transgenic mice, and various window chamber models. Then, we discuss the elucidation of new phenomena and mechanisms relating to tumor immunotherapy that has been made possible by the application of in vivo imaging technology. Specifically, in vivo imaging has supported the characterization of the movement of T cells during immune checkpoint inhibitor therapy and the kinetic analysis of dendritic cell migration in tumor vaccine therapy. Finally, we provide a perspective on the challenges and future research directions for the use of in vivo imaging technology in cancer immunotherapy.

## 1. Introduction

**Figure 1:**
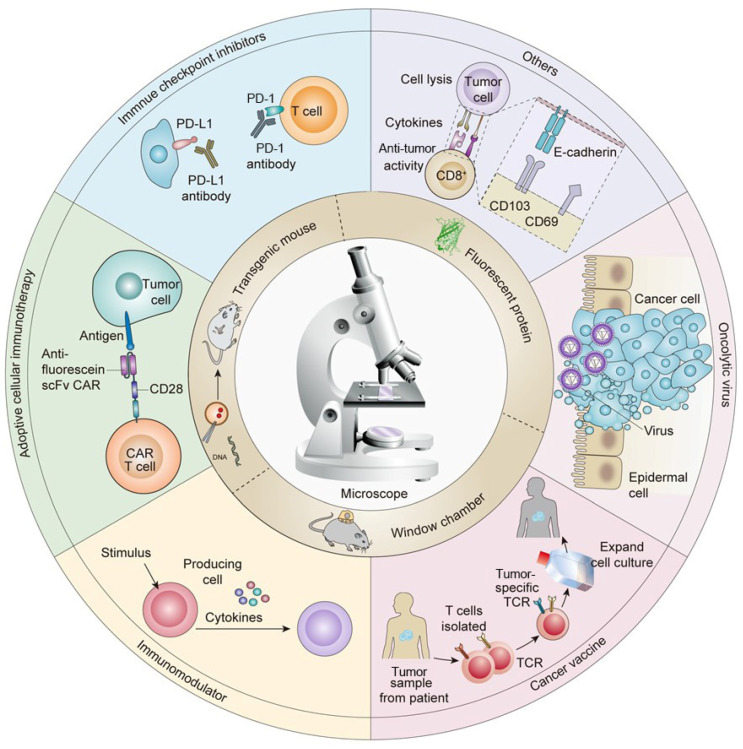
Applications of in vivo imaging techniques for the development of tumor immunotherapies.

At present, immunotherapy is one of the most effective therapies for treating tumors. However, only a minority of patients benefit from immunotherapy. Therefore, to improve the clinical application of immunotherapy, current efforts seek to attain a greater mechanistic understanding of immunotherapy, which has deepened as a variety of novel immunotherapy strategies have emerged. For example, current immunotherapies include ICIs [1,2,3], adoptive cell therapies [4], tumor-specific vaccines [5], and immunomodulatory [6] and other immunotherapies (Figure 1) [7].

The importance of the immune system in the context of cancer was first recognized by Ehrlich in 1904, when he proposed that the immune system may have anti-tumor effects. This idea eventually led to the development of immunotherapy, bringing on a new era of cancer therapeutics. Indeed, the 2018 Nobel Prize in Physiology and Medicine was for the discovery of immune checkpoint blockade [8]. The mechanistic principle of tumor immunotherapy is briefly described as follows. In the early stage, tumor tissue shows invasive growth and causes minor damage to induce inflammatory signals, resulting in the recruitment of immune cells to infiltrate the tumor tissues and secrete IFN-γ [9]. IFN-γ can induce tumor cell death and trigger the release of a large number of cytokines, such as CXCL9, CXCL10, and CXCL11, thereby further recruiting NK cells and macrophages to the tumor microenvironment and inducing a non-specific anti-tumor immune response [10]. In the advanced stage, the tumor cell fragments, which are left over after cell death, are phagocytosed by DCs in the tumor microenvironment, which secrete different chemokines and migrate to the draining lymph nodes [11]. Finally, the DCs present antigens to the T cells, which produce a large number of tumor antigen-specific CD4^+^ T cells and CD8^+^ T cells that specifically recognize and kill tumor cells [12,13].

However, the development of immunotherapies is challenged by the remarkable complexity of interactions between the immune system and tumor microenvironment [14,15]. The tumor microenvironment is composed of tumor cells, mesenchymal cells, tumor micro-vessels, and infiltrating immune cells, all of which interact and show complex fluctuations in composition, phenotype, and movement during the process of tumor immunotherapy [16]. Importantly, the cell type, function, and number of immune cells as well as their cytokine secretion in the tumor microenvironment have implications for the efficacy of anti-tumor immunotherapy [17]. The two main cell types in the tumor microenvironment are both immunosuppressive: Tregs and MDSCs [18,19]. Tregs can inhibit the function of CTLs by secreting transforming growth factor beta (TGF-β) [20], while MDSCs can exert immunosuppressive effects by secreting inhibitory factors, such as prostaglandin E2 [21,22]. In addition, the accumulation of other immunosuppressive factors in tumor tissue, such as IL-1 and IL-10, can also weaken the effects of anti-tumor immunotherapy [23,24]. Moreover, tumor cells can also induce the abnormal functioning of lymphocytes or their apoptosis by secreting inhibitory cytokines, such as DcR3 [25], PD-L1 [26], and FasL [27]. Therefore, understanding the mechanisms by which various immune cells influence the anti-tumor immune response may improve the efficacy of immunotherapy.

In recent years, the emergence of many new technologies has played an important role in uncovering the molecular mechanisms associated with immunotherapy. For instance, flow cytometry allows the characterization of the function and composition of infiltrating immune cell populations [28]; ELISAs facilitate analysis of the type and number of cytokines in the immune microenvironment [29]; WB reveals changes in protein expression in cells [30]; and immunohistochemistry permits visualization of the distribution of immune cells in the tumor microenvironment [31]. These techniques can be employed to determine the changes in cells and the microenvironment in response to immunotherapy and thus provide insight into the mechanisms involved. Importantly, such results support innovations in immunotherapeutic strategies that optimize treatment efficacy. However, there is still room for improvement in both the scope and efficacy of current immunotherapies. Therefore, a deeper understanding of the dynamic changes in the tumor microenvironment during anti-tumor immunotherapy is required to facilitate therapeutic improvements.

In vivo imaging is a single-cell imaging technique for the identification and visualization of molecular targets. In vivo imaging can be employed to observe changes in the number, type, phenotype, and movement of the immune cells during tumor immunotherapy [32,33,34]. In recent years, in vivo imaging has helped researchers explain many new phenomena and mechanisms, which are of great significance in understanding the mechanisms of anti-tumor immunotherapy and improving tumor immunotherapy strategies. In this review, in vivo imaging techniques and methods are introduced and their applications in various immunotherapy strategies are summarized with a particular focus on novel mechanisms and phenomena discovered using in vivo imaging techniques. Finally, current challenges are outlined, and the future development prospects of in vivo imaging and immunotherapy in clinical application are also discussed.

## 2. Technical Methods of In Vivo Imaging

As a rapidly developing biomedical research discipline, in vivo imaging is a powerful technique for achieving visual representation and quantitative analysis at both the cellular and subcellular levels in vivo [35]. The optical microscope is the most basic piece of equipment used for in vivo imaging, standard fluorescence microscopy is commonly used for imaging tissue sections and immunohistochemical imaging to determine whether tissue is diseased and to determine cancer type, but the laser scanning confocal microscope and two-photon microscope are the two most commonly used high-spatio-temporal resolution microscopes for in vivo imaging [36,37]. Laser scanning confocal microscopes can obtain high-quality images by using point scanning and line scanning strategies and is mainly used to observe the details of cell–cell interactions and related three-dimensional distributions [38]. To achieve the increased imaging speed required to visualize living cells, spin-disk confocal microscopy was developed to significantly improve imaging speed while obtaining high-quality images. In contrast to conventional single-point confocal scanning imaging, spin-disk confocal microscopy uses a multi-point simultaneous scanning mode, centered around the Nipkow turntable. Spin-disk confocal microscopy is particularly useful for imaging organs affected by respiratory motion and heartbeat jitter. The two-photon microscope produces high-quality images with limited sample damage by minimizing photon absorption, background fluorescence outside the focal plane, and photo bleaching, while improving the longitudinal resolution; therefore, it is highly valuable for deep tissue imaging in vivo [39]. These three microscopes can obtain real-time, longitudinal, and three-dimensional image information, allowing the visualization of the dynamic behaviors of different immune cells in a specific tissue region, including data on morphology, movement, migration, and cell–cell interactions. This type of high-resolution spatio-temporal dynamic information can shed light on multi-cell participation in key events in the process of an immune response. Therefore, advances in microscopy for in vivo imaging have greatly enhanced our understanding of the immune response.

Furthermore, label-free imaging allows for tumor metabolic assays to detect metabolic abnormalities and tumor growth progression in real time. Minfeng Yang et al. established a label-free metabolic intravital imaging (LMII) technique to detect two-photon excited autofluorescence signals from two coenzymes, NAD(P)H (reduced nicotinamide adenine dinucleotide (phosphate) hydrogen) and FAD (flavin adenine dinucleotide), as robust imaging markers to monitor metabolic responses to immunotherapy [40]. The two-photon fluorescence microscope developed by Wenxuan Liang et al. utilizes the spontaneous fluorescence of NADH for imaging and is capable of tracking the life dynamics of cultured cancer cells and apoptosis-inducing mouse subcutaneous tumor models. The complementary structural and metabolic information provided by this system promises functional histological imaging of unlabeled organs in vivo and in situ, which is expected to be available for clinical diagnosis and therapy [41]. Based on the principle of different wavelengths of light absorption of hemoglobin and deoxyhemoglobin, Deng et al. obtained the structure of mouse liver lobules as well as the functional and structural information of tumor tissues in the diseased areas of liver tumors by photoacoustic microimaging of unlabeled mouse liver tumor regions [42]. Peng Si et al. describe that label-free optical coherency tomography technology has been applied by advanced novel optical designs and algorithms, enabling optical coherency tomography to detect tumor margins and blood vessels more accurately [43]. Jon-Vidar Gaustad et al. showed that a dorsal window model in mice allowed simultaneous MRI imaging to reflect vascular morphology and function within the tumor microenvironment [44].

In addition to advances in microscopy, the development of fluorescent labeling and window chamber technologies have further expanded the scope of application of in vivo imaging technology in anti-tumor immunotherapy [45,46]. At present, gene transfection is the most common approach for inserting a fluorescent label into cells. This technique allows the stable expression of fluorescent proteins in tumor cells or immune cells [47,48]. A variety of fluorescent protein-labeled models have been established to study tumors in mice. For example, Hoffman et al. used a GFP-expressing tumor cell line to study the tumor microenvironment in humanized mice [49,50]. Li Q et al. established a liver metastasis model of pancreatic cancer with stable EGFP expression, which allowed the visualization of liver metastasis in vivo and became an effective tool for assessing pancreatic cancer treatments [51]. Meanwhile, window chamber techniques have also been developed for various organs, such as skin, lung, breast fat pad, abdomen, lymph nodes, bone marrow, and spinal cord, to further deepen our understanding of tumor immunotherapy in vivo [52,53]. The window chamber generally consists of a titanium alloy ring, a thin (approximately 0.3 mmm thick) semicircular resin or glass sheet, or a combination of titanium alloy parts fitted together and surgically inlaid on the outer surface of the imaged tissue, which allow for prolonged microscopic optical observation. For example, Dewhirst MW et al. observed dynamic changes in tumor tissue and blood vessels by transplanting tumor cells into a dorsal spinal window chamber [54,55]. Similarly, the intracranial skin window chamber model has been used to study the effects of drugs on intracranial tumors and investigate the role of bone marrow-derived cells in the angiogenesis of brain tumors [56]. Another study by Shan S et al. used a rodent breast window chamber for longitudinal observation of the vascularization and blood flow changes in the tumor microenvironment, which led to their proposal of various treatment strategies of breast cancer [57]. To study lung cancer, Hariri et al. used a lung window chamber model to investigate cell–cell interactions, membrane dynamics, and vascular perfusion in the early stage of lung cancer metastasis [42]. In another example, Haessler U et al. evaluated the morphological characteristics and regeneration kinetics of lymph nodes using a skin window chamber model [58]. Meijer et al. also studied lymph nodes, observing the activation and function of immune cells in lymph nodes by using a lymph node window chamber model [59]. Window chamber models are also effective for studying abdominal organs. For example, Deng et al. designed a drawer-type abdominal window chamber model, permitting the long-term fluorescence/photoacoustic bimodal microscopic imaging of living liver tissues [42]. Moreover, Chuprin J et al. clarified the mechanism of regenerative therapy in acute renal injury by using an embedded abdominal window chamber. Therefore, as evidenced by its effective use to study many different tissue types, window chamber models are an important technique for exploring the dynamic changes in cells during tumor treatment [3,60]. However, limitations include the trauma associated with the window chamber and species-specific differences. Therefore, window chamber models should be considered in conjunction with humanized mouse models. Humanized mouse models, as described above, offer an important alternative to overcome the problem of the differences between mice and humans, and recipient mice can even be genetically engineered to study specific immune system attributes [60,61]. When tumor tissues from patients are transplanted in situ or ectopically, dynamic and longitudinal information can be obtained in relation to the immune response to human antigens. Moreover, humanized mice can be applied alongside other technologies, such as in vitro organoid culture and spontaneous tumor mouse models, to perform in vivo imaging to answer specific experimental questions [62,63].

Most importantly, in addition to observing correlations between immune cells and other cells during the process of immunotherapy for various tumors, in vivo imaging technology also visually and quantitatively describes the dynamic behavior of immune cells, including morphological changes, phagocytosis, or other important events [64]. Quantitative data collected by in vivo imaging can be used for statistical analysis to better characterize the tumor microenvironment and immune cell responses to tumor immunotherapy [65]. For example, observing the dynamic behavior of DCs and T cells after antigen uptake using in vivo imaging has been used to evaluate the antigen presentation ability of DCs, and recording the interaction time between effector T cells and tumor cells can be indicative of effector T cell function [66]. In conclusion, in vivo imaging is an indispensable technical means for promoting the development of cancer immunotherapy. The summarized in vivo imaging window types and transgenic fluorescent protein mouse species are shown in Table 1.

## 3. Applications of In Vivo Imaging in Studying Tumor Immunotherapy

The purpose of tumor immunotherapy is to activate or enhance the body’s own immune system and destroy the tumor cells through the action of CD8^+^ T cells [86,87]. Immunotherapies have become a first-line treatment option for several cancers and have been widely used in combination with surgery [88], radiotherapy [89], chemotherapy [90], targeted therapy, or other cancer treatments [91,92]. At present, tumor immunotherapies include ICI therapy [93], immunomodulatory therapy [94], tumor vaccines [95], oncolytic viruses [96], and adoptive cell therapy [97], all of which have been approved or are in the clinical evaluation stage and have achieved excellent therapeutic effects [98,99]. However, exploring the mechanisms involved in tumor immunotherapy remains an important priority. Given its powerful ability to visualize cell-level events over time, in vivo imaging technologies have great value for studying the dynamics of immune cells during tumor immunotherapy [100]. For example, Mempel TR et al. demonstrated the interactions between lymphocyte migration, DCs, and cancer cells in anesthetized mice using a two-photon microscope [101]. Furthermore, in vivo imaging technologies can be used to observe new phenomena and mechanisms across different types of immunotherapies; these advances are discussed in detail in the latter part of this review.

### 3.1. Applications of In Vivo Imaging in the Study of ICI Therapy

**Figure 2:**
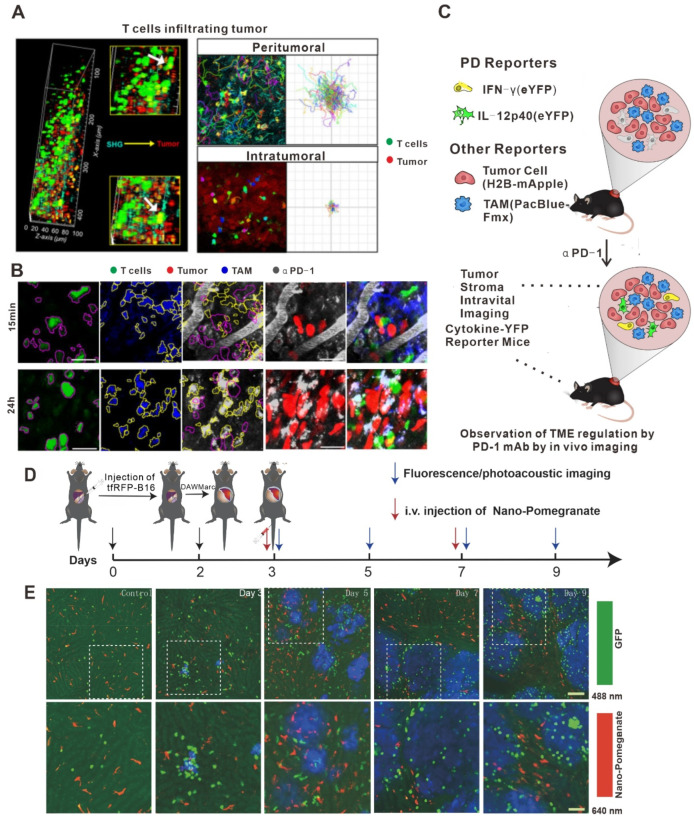
Application of in vivo imaging in the study of immune checkpoint inhibitors. (**A**) Intravital imaging was performed using multiphoton microscopy with second harmonic generation to detect the stroma–tumor interface and CD8^+^ GFP^+^ T cells infiltrating tumors at a tissue penetration depth of 100 µm. Track plots, velocities, and meandering indices of T cells were measured from T cells located separately within the peritumoral and intra-tumoral regions. Micrographs of T cells (magenta outline) identified as GFP^+^ cells and TAMs (yellow outline) identified by Pacific Blue. Outlines are overlaid on micrographs of the corresponding AF647–aPD-1 channel. Scale bars, 30 mm. (**B**) Z-projections of an MC38–H2B-mApple tumor in a DPE-GFP mouse injected intravenously with AF647–aPD-1 after 15 min (top) or 24 h (bottom). (**C**) Schematic photo of MC38 tumors in IFN-g-eYFP reporter mice treated or not with aPD-1 mAb. Yellow, IFN-g-eYFP-expressing cells; red, tumor cells; and blue, PacificBlueFMX-labeled tumor-associated macrophages (TAMs). (**D**) Long-term intravital imaging of the microenvironment surrounding liver metastases. Schematics of the procedures and timeline of tfRFP-B16 cell injection, surgery for implantation of DAW, and long-term fluorescence confocal imaging of liver tfRFP-B16 metastases. (**E**) Intravital fluorescence confocal imaging of liver metastases microenvironment at different times. Blue: tfRFP-B16 tumor metastases; green: GFP-labeled NKT cellks; and red: Nano-Pomegranate-labeled Kupffer cells. Top row: 2 × 2 large-field images; scale bar: 100 µm. Bottom row: images of the region of interest from the top row; scale bar: 50 µm. Copyright 2020 The Author(s). (**A**) adapted from Ref. [80], under a CC BY license. (**B**,**C**) adapted from Ref. [106] (**D**,**E**) adapted from Ref. [42] under a CC BY license, link of the license: https://creativecommons.org/licenses/by/4.0/).

Targets of ICIs include LAG-3, CD40, GITR, CD137, CEA-TCB, OX40, PD-L1, and CTLA-4 [102,103]. The 2018 Nobel Prize in Physiology or Medicine was awarded to the two scientists who discovered PD-1 and CTLA-4. Inhibitors of PD-1 (or its ligand, PD-L1) and CTLA-4 are two of the most successful classes of clinical immunosuppressive therapies [102]. However, ICI therapy has different effective response rates in different tumor types: the highest effective rate of ICI therapy could reach 50% (in melanoma and MSI-H tumors), while in gliomas, the effective response rate was less than 10% [104]. It was previously suggested that this phenomenon was due to the instability of the tumor microenvironment and the mutation load. Recently, in vivo imaging techniques provided new evidence to suggest that this phenomenon did not apply to all clinical samples [105]. A study in a mouse melanoma model by Lau D et al. used in vivo imaging to clarify the changes in CD8^+^ T cell migration and morphology before and after treatment with an antibody against PD-L1. As is shown in Figure 2A, many CD8^+^ T cells at the edge of the tumor show Lévy-like movement before anti-PD-L1 injection with a significantly higher movement rate than those near the tumor tissue, a pattern of migration that maximizes the chance of encounters with their target tumor cells. After injection of the PD-L1 antibody, the number of T cells in tumor tissue increased and their movement rate decreased significantly [80]. Hence, this study illustrates the value of in vivo imaging technology in elucidating the dynamic behavior of T cells during anti-PD-L1 treatment. In another example, in vivo imaging was used by Arlauckas SP et al. to discover that the Fc receptor on the surface of myeloid cells was a key factor affecting the therapeutic effect of PD-1 inhibition [81]. They found that the PD-1 antibody transferred from T cells to the surface of PD-1-negative tumor-associated macrophages within a few minutes after the PD-1 antibody bound to PD-1. This binding occurs between the glycosylated region of the Fc segment of the PD-1 antibody and the Fc receptor on the surface of myeloid cells. Moreover, the therapeutic effects of ICIs were significantly improved after blocking the Fc receptor, indicating that the Fc receptor is likely a key reason behind the low response rate of PD-1 inhibitor treatment (Figure 2B). Garris CS et al. also found that tumor-infiltrating DCs, a class of immune cells that do not respond to PD-1 antibodies, could promote the therapeutic effects of ICIs by responding to the IFN-γ signals released by neighboring T cells and releasing cytokines such as IL-12 (Figure 2C) [106]. Therefore, in vivo imaging technology has the potential to aid in the discovery of more new phenomena relating to ICI therapy in the future.

### 3.2. Applications of In Vivo Imaging in Characterizing Immunomodulator Therapy

Cytokine therapy [107], chemokine therapy [108], and small molecule inhibitor therapy are commonly used immunomodulatory therapies [109,110] that seek to treat cancer by improving the tumor-targeting abilities of the immune system. Immunomodulatory therapies target both innate and adaptive immune cells by activating NK cells [111], macrophages [112], and effector T cells [22] and inhibiting MDSCs and Tregs [113,114]. As for ICI therapies described above, in vivo imaging technologies are also valuable for better understanding the mechanisms by which immunomodulatory therapies impact immune cells, thus providing guidance to further optimize treatment strategies.

**Figure 3:**
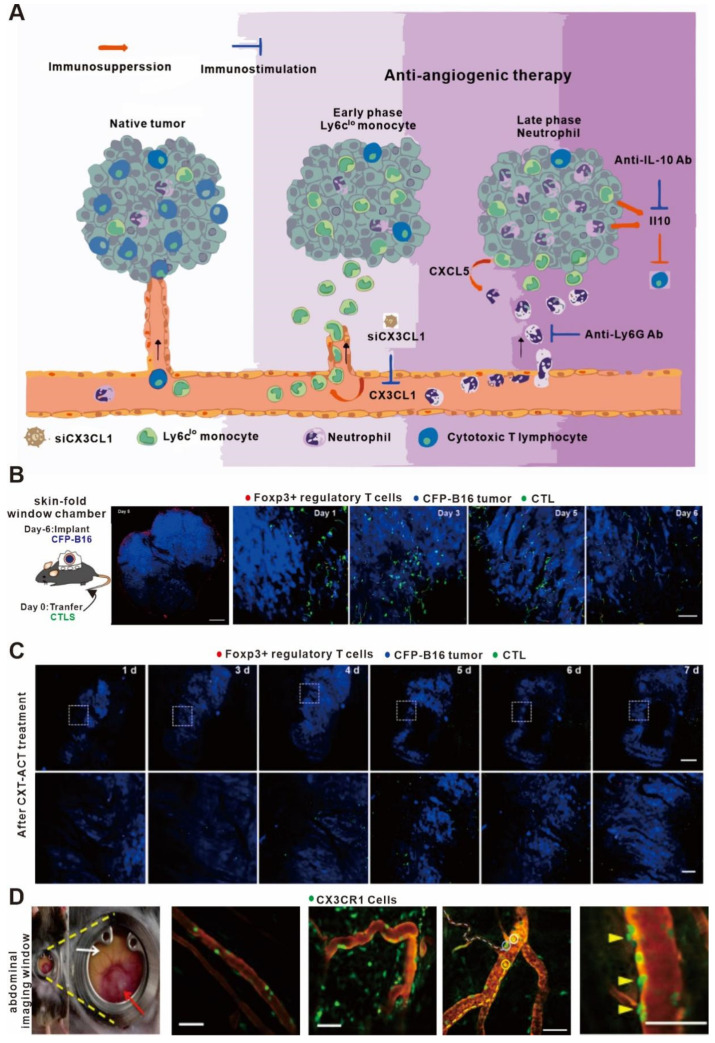
In vivo imaging applications in studies on immunomodulators. (**A**) Proposed mechanism of antiangiogenic therapy-induced immunosuppression. (**B**) Large-field intravital images of an ‘immunosuppressive ring’ around the CFP-B16 tumor. Blue—CFP-B16 tumor; red—Tregs (Foxp3-mRFP cells); and green—CFSE-labeled CTLs. The left panel shows different single-color channels of the tumor microenvironment, and the right panel shows the three color channels merged. Scale bar: 500 µm. (**C**) Long-term intravital imaging of the multicolor-coded tumor environment in CTX-ACT-treated mice. Red—Tregs (Foxp3-mRFP); green—CSFE-labeled CTLs; and blue—CFP-B16 tumor. Top row: large-field images; scale bar: 500 µm. Bottom row: images from the region of interest in the top row; scale bar: 100 µm. The imaging data are representative of similar results from 3–5 mice in two independent experiments. (**D**) Abdominal imaging window on a live mouse bearing syngeneic SL4 CRC (red arrow) in the cecum (white arrow). Images of crawling CX3CR1^+^ leukocytes (green) inside the post-capillary venule (red, TRITC-dextran) in a normal cecum and in the tumor of a CX3CR1 GFP/^+^ mouse. Ly6Clo monocytes are labeled with EGFP (green). (**A**) adapted from Ref. [83] (**B**–**D**) adapted from Ref. [82] under a CC BY license; and link of the license: https://creativecommons.org/licenses/by/4.0/).

Treg cells are an essential type of immunosuppressive cell in the body that inhibit the function of effector T cells [115,116,117]. In clinics, cyclophosphamide chemotherapeutic drugs are frequently used to inhibit the Treg cells to promote anti-tumor immunity [118]. In order to explore the mechanistic properties of Treg cells after the addition of these drugs, Qi S et al. constructed a fluorescent-labeled transgenic mouse and skin window chamber model for observing the dynamics of Treg cells using in vivo imaging technology [82]. As shown in Figure 3A, anti-vascular endothelial growth factor receptor 2 therapy upregulates CX3CL1 expression, which attracts CX3CR1+Ly6Clo monocytes (middle, early stage), followed by neutrophils via CXCL5 (right, late stage), resulting in an immunosuppressive microenvironment and a reduction in cytotoxic T lymphocytes in the tumor. This multistep process provides multiple points of intervention to prevent immune resistance and enhance the efficacy of anti-vascular endothelial growth factor therapy [83]. As shown in Figure 3B,C, they found that the Treg cells could form an immunosuppressive ring around the tumor in order to encapsulate the effector T cells that infiltrated the tumor tissue, thereby affecting the efficacy of anti-tumor immunity. When cyclophosphamide was added, it destroyed the immunosuppressive circle and successfully increased the infiltration of effector T cells in tumor tissues, thus illustrating a key mechanism of this immunotherapy.

Anti-VEGF drugs had long been thought to promote tumor immunotherapy by promoting vascular normalization [119,120]. However, Goel S et al. showed that the addition of VEGF suppressed the effects of immunotherapy in a colorectal cancer model using in vivo imaging technology. As shown in Figure 3D, they observed that the anti-VEGF drugs not only increased the content of CX3CL1 and CXCL5 in tumor vessels but also promoted the recruitment of neutrophils, resulting in the release of a large amount of the cytokine IL-10 to inhibit the adaptive immunity [121]. In addition, Jung K et al. found that epinephrine could inhibit the movement of immune cells and hinder the effects of anti-tumor immunity by regulating angiogenesis in the tumor microenvironment [83]. Therefore, in vivo imaging technology has been valuable for characterizing the effects of anti-VEGF drugs on the anti-tumor immune process.

Cytokine therapy is also an important immunomodulatory therapy [107]. At present, significant effort has been placed into the study of the anti-tumor therapeutic mechanism of several cytokines, such as IL-2 [122], IL-12 [123], IL-15 [124], IL-24 [125], IFN-γ [126], IL-1β [127], TNF-α [128], GM-CSF [129], and others [130]. For these cytokines, Schumacher’s and Busso’s research groups were the first to show three-dimensional dynamic images of tumor tissues following IFN-γ treatment using in vivo imaging technology [131,132]. They found that IFN-γ not only killed the tumor cells that interacted with T cells but also killed distant tumor cells through the mechanism of the bystander effect. Therefore, the in-depth intersection and development of in vivo imaging technology and immunology has the potential to further reveal new phenomena and mechanisms.

### 3.3. Applications of In Vivo Imaging in Cancer Vaccine Studies

Tumor vaccines are an important immunotherapy that involves stimulating the body to produce a large number of specific memory T cells to target tumor cell antigens and drive tumor cell clearance [95,133]. However, tumor cells possess a strong innate ability to escape immune detection, leading to the development of immune tolerance to auto-antigens and the loss of efficacy of anti-tumor immunotherapies [13]. In vivo imaging technology can assist in observing the dynamic correlations between immune cells and antigen-presenting cells after vaccination, thus contributing to a deeper understanding to overcome immune escape.

**Figure 4:**
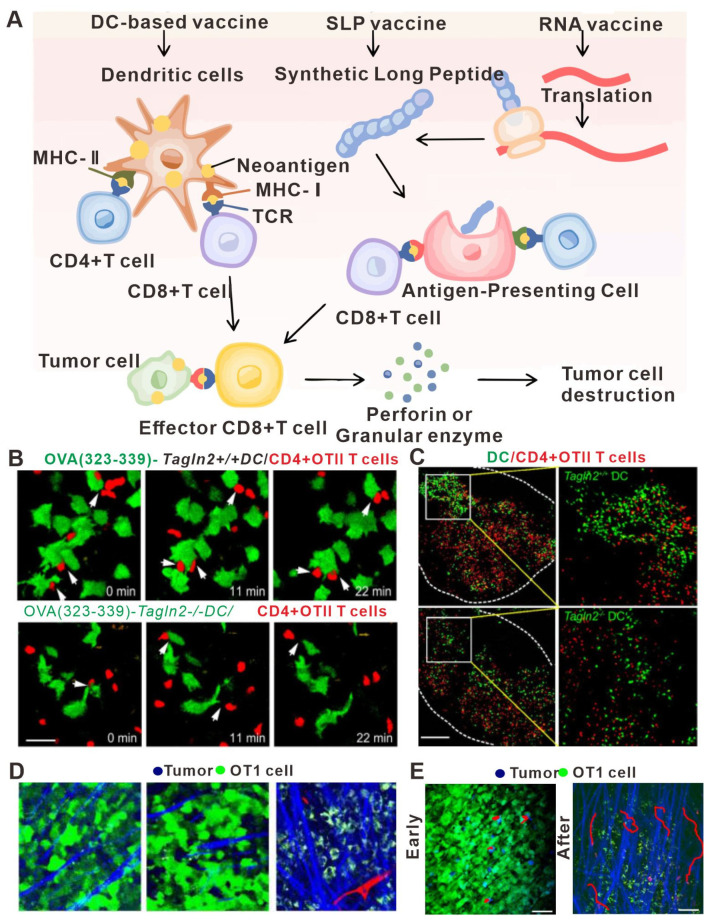
In vivo imaging applications in studies relating to tumor vaccine design. (**A**) Major types of neoantigen vaccine. In vivo, neoantigens are eventually presented to CD4^+^ T cells and CD8^+^ T cells to induce specific immune responses and achieve anti-tumor effects. (**B**) Image of a representative DC–T cell interaction. WT or Tagln2^−/−^ DCs were pulsed with pOVA (323–339) and co-incubated with OTII CD4^+^ T cells for 1 h. Scale bar: 5 μm. (**C**) Representative cryosection images showing the overall distribution of WT or Tagln2^−/−^ DCs (green) and OTII CD4^+^ T cells (red) in draining lymph nodes. Scale bar: 100 μm. (**D**) Representative snapshot of live images of DC–T cell interactions in vivo. Representative TPLSM images (160 × 160 μm) of GFP-EG7 or GFP-EL4 tumors during early (day 3) and late phases (day 6) of tumor rejection after adoptive transfer of 107 OT1 cells. Vessels (red) are imaged by intravenous injection of 70 kD rhodamine-dextran (2.5 μg/mL) and collagen fibers (blue) using SHG signals. Capture parameters were identical for all images. Bar: 44 μm. (**E**) TPLSM images of OT1-CFP cells within EG7-GFP tumors (green) during early phase (day 4) and late phase (day 5) of tumor rejection. Collagen fibers (blue) are imaged by SHG. Examples of typical migratory paths (red) are shown. (**A**) adapted from Ref. [140] (**B**,**C**) adapted from Ref. [137] (**D**,**E**) adapted from Ref. [84] under a CC BY license, link of the license: https://creativecommons.org/licenses/by/4.0/).

DCs are the key cell type that links the innate and adaptive immune systems, thus inducing the humoral and cellular responses against a specific antigen [134]. After taking up the tumor antigen, DCs migrate to the draining lymph nodes and present the antigens to T cells, activating the specific T cells that will recognize the tumor antigen and kill the tumor cells [135]. Recent advances in understanding tumor vaccines have led researchers to suggest that promoting the migration ability of DCs might further improve the efficacy of anti-tumor therapy [136], and in vivo imaging has been used to support these advances. For example, Kim HR et al. discovered an inhibitory role for transgelin-2 in the migration and antigen presentation of DCs in a cancer model using in vivo imaging technology. Knockout of the transgelin-2 gene in DCs resulted in no effect on their maturation and differentiation. However, interestingly, the ability of DCs to migrate to the lymph nodes and form immune synapses was greatly weakened, resulting in the loss of immune recognition of the tumor antigens (Figure 4A–C). Furthermore, the direct incubation of deubiquitinated recombinant transgelin-2 with DCs in vitro further enhanced the antigen presentation and migration ability of DCs and the effects of anti-tumor therapy [137]. Therefore, in vivo imaging technology was used to demonstrate that the transgelin-2 gene can be an important target for the design of DC-targeting vaccines in the future.

Another revolutionary discovery using in vivo imaging technology in the field of tumor vaccines is determining the nature of tumor antigens. For a long time, tumor antigens were thought to exist in the form of peptide antigens [138]. However, using a new fluorescent protein antigen model and in vivo real-time imaging, Yang F et al. showed that the tumor antigens existed mainly in the form of vesicles (Figure 4D,E) [84]. Moreover, in vivo imaging technology has also subverted the traditional understanding of the function of macrophages. Macrophages have always been thought to have no function in antigen presentation. However, using in vivo imaging, Moalli F et al. demonstrated a process in which macrophages present antigens to B cells in lymph nodes [139]. First, tumor cells release antigens to nearby draining lymph nodes in the form of vesicles, which are quickly absorbed by the subdorsal macrophages and transported to follicular DCs. Then, the antigens are recognized by sentinel B cells to produce antigen-specific IgG antibodies. To sum up, in vivo imaging technology has not only helped in identifying potential therapeutic targets that affect DC migration, but also in shedding light on the very nature of tumor antigens, thus potentially providing valuable information to guide the design and optimization of tumor vaccines.

### 3.4. Applications of In Vivo Imaging in Understanding Oncolytic Virus Therapy

Oncolytic virus immunotherapy has emerged as one of the fastest developing immunotherapies and has been used for treating various types of tumors [141]. Oncolytic virus immunotherapy is particularly valuable because it strongly targets and kills tumor cells while sparing normal cells and also improves the tumor microenvironment. The anti-tumor immune mechanism of oncolytic virus immunotherapy includes the following main processes: (1) Oncolytic viruses trigger the release of tumor antigens by destroying the tumor cells, thus inducing the tumor-specific T cell response following antigen uptake by antigen-presenting cells [142]. (2) Oncolytic viruses can induce a variety of immunogenic death modes in tumor cells, such as necrosis, necrotizing apoptosis, and immunogenic apoptosis, causing tumor cells to release a large number of damage-associated molecular patterns (DAMPs) or even release their viral components, thus enhancing the effects [143] of anti-tumor immunotherapy [144]. (3) Specific oncolytic viruses can disturb the blood vessels in tumor tissues and promote immune cell infiltration in the tumor tissue microenvironment [145]. (4) Tumor cells infected with oncolytic virus can release pro-inflammatory factors that cause the tumor microenvironment to be less immunosuppressive [143]. 

**Figure 5:**
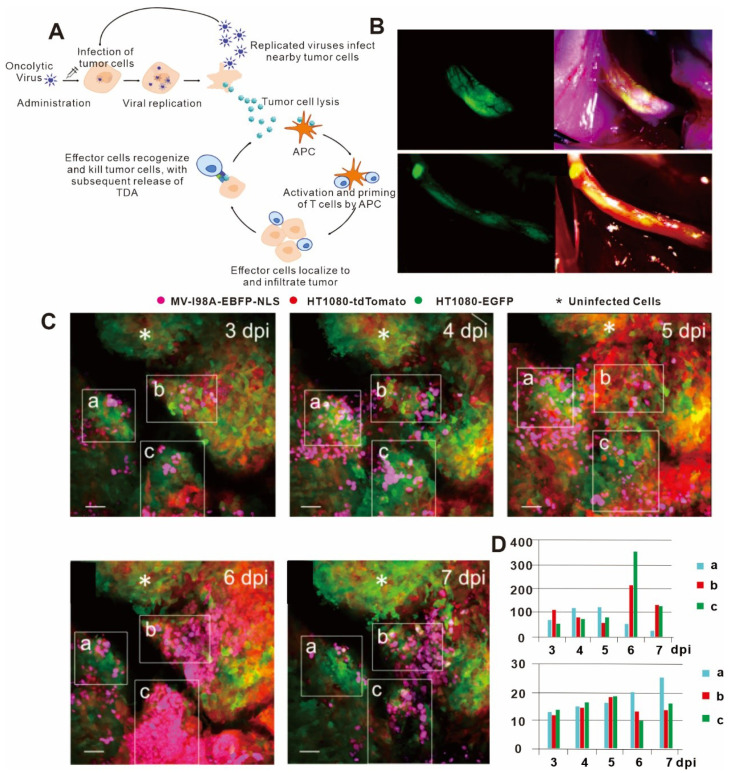
(**A**) Mechanism of action and immunogenic response to oncolytic viruses for use in cancer therapy. Lysis of tumor cells following viral replication results in release of tumor-derived antigens (TDAs), which promote the activity of the cancer-immunity cycle, ultimately resulting in the development of a tumor-specific immune response. APCs = antigen-presenting cells. (**B**) NV1066 lysovirus selectively localizes to nerve-infiltrating regions of prostate and pancreatic cancer cells. Fluorescent image of tumor (left), and tumor physical picture (right). (**C**) Imaris spot analysis software was used to detect infected nuclei in three regions of interest (ROIs), which could be precisely retraced over time at 3 days post infection (dpi), 4 dpi, 5 dpi, 6 dpi, and 7 dpi. White asterisks denote uninfected tumor areas. Scale bars: 50 μm. (**D**) Histogram of number of infected cells and median of minimum distances to closest neighbor over time in ROIs. (**A**) adapted from Ref. [142] (**B**) adapted from Ref. [151]; and (**C**,**D**)citations are from Ref. [149] under a CC BY license, link of the license: https://creativecommons.org/licenses/by/4.0/).

The replication of oncolytic viruses can be considered analogous to the principle of population dynamics in ecology; that is, oncolytic virus replication can be almost perfectly explained using a mathematical model in vitro [146,147]. However, the nature of oncolytic virus replication in vivo is more complex and requires the implementation of in vivo imaging for its characterization. Specifically, in vivo imaging technology can provide longitudinal data on the interactions between tumor cells and oncolytic virus and between the local microenvironment and the immune system, thus allowing correlations to be drawn concerning the tumor, virus, and immune system [148]. For example, Kemler I et al. used in vivo imaging technology to determine the diffusion kinetics of oncolytic virus in the tumor microenvironment [149] (Figure 5B–D). The entry of oncolytic virus into cells by membrane fusion was found to peak within two to three days after intra-tumoral injection in most cases, but cells with low levels of viral membrane fusion peaked at about six days after intra-tumoral injection. The extent of viral membrane fusion was a key predictor of successful oncolytic virus infection, as cells experiencing high levels of membrane fusion had infection rates more than three times higher than those that showed low levels of membrane fusion. Nair M et al. also used in vivo imaging to study oncolytic viruses and found that oncolytic virus infection of blood vessels resulted in activation of vascular endothelial cells, which inhibited virus replication and thereby impaired the killing effect of the oncolytic virus on perivascular tumors [150]. However, when the gene for angiostatin, which has anti-vascular effects, was loaded into the host virus, it could significantly reduce the endothelial cell density in the tumor microenvironment and enhance the anti-tumor immune effect. These results highlighted the importance of considering the role of blood vessels when assessing the killing efficacy of oncolytic virus on tumor cells in vivo.

In summary, in vivo imaging technology can directly observe the interaction of oncolytic virus with tumor and immune cells, thereby enabling researchers to further optimize treatment strategies.

### 3.5. Applications of In Vivo Imaging in Studies on Adoptive Cell Therapy

Adoptive cell therapy is a form of immunotherapy that involves the transfer of anti-tumor immune cells (specific or non-specific) into the patient to directly kill the tumor cells or stimulate the immune response [4,152,153]. Perhaps the most well-known form of adoptive cell therapy is CAR-T cell therapy. In 2012, CAR-T cell therapy successfully induced remission in a leukemia patient who received a transfusion of genetically modified CAR-T cells, making it currently the most effective adoptive cell therapy [154]. CAR-T cells can identify tumor cells independently of the expression of MHC [155]. However, the therapeutic effect of adoptive cell therapy in solid tumors remains inferior to that in hematological cancers [156]. At present, the immunosuppressive microenvironment and complex tumor tissue composition in solid tumors are the main reasons for the limited efficacy of CAR-T cell therapy for this type of cancer [157,158,159].

**Figure 6:**
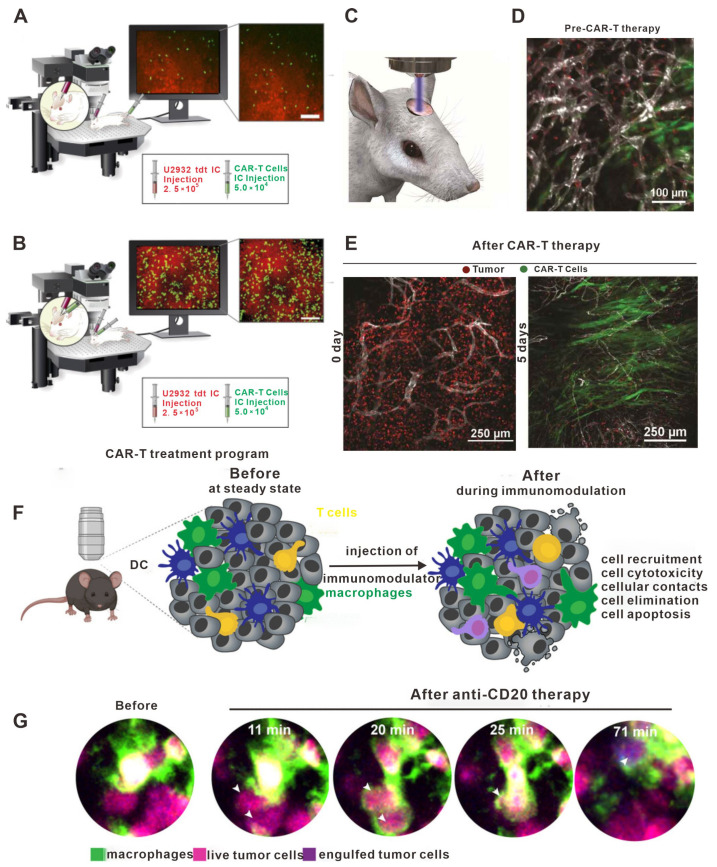
The role of in vivo imaging in studies on adoptive cell transfer. (**A**) Intravenous administration resulted in a low presence (infiltration, accumulation, and depth) of h19m28z CAR-T cells (targeting CD19) without a sustained effect on tumor cells in the majority of mice. (**B**) In contrast, after intracerebral injection, h19m28z CAR-T cells were present at higher numbers and higher depths, compared with mock CAR-T cells. h19m28z CAR-T cells also had low velocity, compared with mock CAR-T cells, owing to immune synapse formation and the killing of tumor cells, leading to reduced primary central nervous system lymphoma. (**C**–**E**) Visual schematic of intravital imaging shows the physical orientation of dynamic and longitudinal in vivo monitoring via a surgically implanted window chamber. Intravital imaging 24 h after treatment demonstrated CAR-T cell extravasation from the vasculature following whole body irradiation. Intravital imaging of tumor-bearing mice 5 days following CAR-T cells revealed expansive CAR-T cell proliferation and corresponding tumor regression. Imaging tumor-bearing mice 5 days following CAR-T cell treatment without WBI revealed inferior penetration within the tumor bed, leading to a suboptimal therapeutic response as compared to CAR-T cells and WBI treatment. (**F**) Confocal microscopy provides a unique opportunity to observe the immediate effects of immunomodulators in real time. Confocal microscopy allows imaging of the exact same location immediately before and immediately after the injection of an immunomodulator. This is particularly useful for characterizing the early effects of immune interventions on cellular behavior. DCs represents dendritic cells. (**G**) Example of bone marrow images of B cell tumor-bearing mice acquired by confocal microscopy before and a few minutes after injection of anti-CD20mAb. Macrophages stained with anti-F4/80 antibody are shown in green. Live tumor cells are shown in magenta, then turn blue upon macrophage phagocytosis. White arrows highlight phagocytosed tumor cells. (**A**,**B**) Adapted from Ref. [165]; (**C**–**E**) adapted from Ref. [161]; and (**F**,**G**) adapted from Ref. [166] under a CC BY license, link of the license: https://creativecommons.org/licenses/by/4.0/.

Therefore, the use of in vivo imaging to observe CAR-T cell migration in solid tumors is likely to be highly valuable for understanding the mechanisms that occur during adoptive cell therapy. Indeed, Mulazzani M et al. studied the movement of anti-CD19 CAR-T cells and non-targeted CAR-T cells in solid tumors in vivo using the cranial window chamber model [159]. Their results showed that the infiltration of both types of CAR-T cells into the intracranial tumors was similar after tail vein injection. However, when injected into the brain, the anti-CD19 CAR-T cells penetrated more deeply into the tumor parenchyma, while the non-targeted CAR-T cells remained primarily localized on the periphery of the tumor. Flow analysis data showed that the expression of CD27 on the T cell surface in the anti-CD19 CAR-T cells was significantly lower compared to the non-targeted CAR-T cells, which were associated with significantly longer survival time compared to non-targeted CAR-T cells (Figure 6A,B). These results suggested that the modification of CAR-T cells to target certain molecules may not affect cell migration but does impact the lifespan of the cell and its ability to infiltrate tumor tissue. Moreover, in vivo imaging has also been used by Mastereo Y et al. to show that the number of effector T cells around blood vessels was significantly higher than those in hypoxic tumor tissues, indicating that the normalization of tumor vessels may be another approach to enhance the efficacy of CAR-T cell therapy [160].

Other studies have provided important insights into the use of more traditional therapies alongside CAR-T cell therapy. Murty S et al. reported for the first time that radiotherapy could improve the ability of CAR-T cells to invade solid tumors (Figure 6F,G) [161]. Ito F et al. found that local tumor hyperthermia could enhance the expression of ICAM-1 in the tumor vasculature, thus promoting the infiltration of CAR-T cells; these results suggest that physically destroying the solid tumor microenvironment may promote the therapeutic effects of CAR-T cells [162]. However, as normal tissues also express CAR-T targets, it is critical to consider the possibility of autoimmune diseases, which is an important limitation of CAR-T therapy in solid tumors [163]. As shown in Figure 6D,E, Tucker CG et al. observe differences in the efficacy of CAR-T cell therapy when comparing cells with different affinities using in vivo imaging technology, finding that increased expression of TAA in tumor cells may weaken the affinity of CAR-T cells for target tumor cells [164]. Hence, when designing CAR-T cell therapy for tumors that express high levels of TAA, selecting cells with low affinity may mitigate autoimmune diseases without compromising the therapeutic effect.

### 3.6. Other Applications of In Vivo Imaging for Studying Anti-Tumor Immunity

In addition to the types of anti-tumor immunotherapy described above, there are a variety of other forms of immunotherapy under development that involve a range of immune cell types. For example, neutrophils are also important for anti-tumor immunotherapy, but mechanisms that promote or inhibit neutrophil activity during tumor immunotherapy are not yet clear [167]. Teijeira A et al. used in vivo imaging technology to describe the processes by which neutrophils promote tumor cell metastasis and immune evasion. Specifically, tumor cells could produce chemokines, which activated CXCR1 and CXCR2 receptors on the surface of neutrophils to result in the formation of NETs, which surround tumor cells and protect them from direct contact with cytotoxic CD8^+^ T cells and NK cells [168]. Furthermore, the efficacy of immune checkpoint inhibitors was further enhanced when four inhibitors of protein arginine deaminase were used to inhibit NET formation. 

In the past, the MDSCs were generally regarded as a type of immunosuppressive cell [169]. However, Liu TW et al. found that these cells could inhibit tumor growth and promote T cell activation [170]. They used in vivo imaging techniques to confirm that MDSC activation produced phagosomes containing peroxidase and catalyzed the production of substances such as hypochlorite, which directly inhibited the activity of I-kinase B kinase in the tumor cells and promoted tumor cell apoptosis. In addition, hypochlorite could also induce changes in the CD8^+^ T cell transcriptome and activate mitogen-activated protein kinase (MAPK) and phosphatidylinositol 3-kinase/protein kinase B (PI3K/AKT) signaling pathways to promote T cell activation.

Recently, the role of tissue-resident cells in anti-tumor immunity has also attracted the attention of researchers [171,172]. Park SL et al. found that epidermal-resident memory T cells played a key role in preventing epidermal tumor development and recurrence [173]. Using in vivo imaging techniques in mouse melanoma models, they found that many tumor-specific epidermal CD69^+^ CD103^+^ TRM T cells increased over time and gradually killed the tumor cells. NK cells are also an important factor, affecting the efficacy of anti-tumor immunotherapy [174]. Similarly, Liu L et al. used in vivo imaging techniques to show that the liver microenvironment could affect the efficacy and timing with which effector T cells killed tumors [175]. In the liver microenvironment, the activation of sinusoidal endothelial cells with melittin nanoparticles, which promoted the release of CXCL9 and CXCL10, induced the recruitment of a large number of NK cells into the liver in order to eliminate tumors. In addition, in vivo imaging techniques are also valuable for evaluating the formation of tumor-specific immunological memory. Qi S et al. first used photothermal therapy to treat B16 melanoma-bearing mice and then re-inoculated them with homologous tumor cells [79]. The whole process of memory T cell differentiation and effector T cell attack of the re-inoculated tumor cells was recorded using in vivo imaging techniques. In conclusion, in vivo imaging technologies are an important technical aid for analyzing the processes and mechanisms that occur with the implementation of a novel immunotherapy strategy. The in-depth, longitudinal data acquired can highlight important details regarding immune cell function and provide guidance for the further understanding of the mechanisms of immunotherapy.

## 4. Discussion

The efficacy of immunotherapy is intricately tied to the movements and functions of immune cells in vivo. Therefore, in vivo imaging technologies, which can dynamically observe events such as immune cell aggregation, migration, and cellular contact time, have greatly helped in the discovery of many new phenomena that occur regarding immune cells during immunotherapy. In this review, the techniques related to the application of in vivo imaging were first introduced. Then, new mechanisms and phenomena relating to immunotherapy that were discovered by in vivo imaging were discussed, with particular focus on those ignored in the past. In vivo imaging provides a large amount of data, which are valuable for the formulation and optimization of tumor immunotherapy strategies. In the future, further developments in the field of in vivo imaging could allow for greater insight into dynamic changes in the molecular activation of real-time signaling pathways. This is because the current image resolution is not sufficient to study the molecular dynamics of signaling pathways. Improvements in spatial resolution and temporal resolution in the process of real-time dynamic imaging will be a major challenge for the use of in vivo imaging technology in the future. In addition, another challenge is the acquisition of information relating to immune function at great tissue depths. Future studies should focus on the combination of optical fiber probes and in vivo imaging technology in order to interrogate the function of immune cells and tumor cells in less accessible organs, such as the intestines, stomach, pancreas, and other organs located more deeply in the body. Finally, the combination of single-cell sequencing, spatial transcriptomics, mass spectrometry, and clustered regularly interspaced short palindromic repeat-associated nuclease 9 (CRISPR/Cas9) technology with in vivo imaging may provide more insights into the mechanism of immunotherapy and hopefully improve treatment outcomes in clinical settings.

## Figures and Tables

**Table 1:** Existing in vivo imaging techniques and methods.

	Type	Application	Ref.
Optical microscope	Laser scanning confocal microscope	Laser scanning confocal microscopy confirmed the expression of CD19 and CD3 proteins in lymphocytes	[67]
	Two-photon microscope	Deep imaging of tumor tissue by two-photon microscope	[68]
		Spinning-disk confocal microscopy improves image quality by preventing pinhole cross-talk forintravital imaging	[69]
Window chamber	Dorsal	Direct observation of blood vessels around a tumor	[69]
	Intracranial	Observation of the effect of drugs on intracranial structure	[70]
	Breast	Dynamic observation of blood vessels and blood flow	[57]
	Liver	Observation of dynamic changes among immune cells in liver	[42]
	Lung	Observation of dynamic changes in the microenvironment before lung metastasis	[71]
	Skin	Observation of dynamic changes in the lymph node microenvironment	[72]
	Abdomen	Analysis of cell function in acute kidney injury and clarification of the mechanisms of regenerative therapy	[73]
	Lymph node	Observation of the dynamic behavior of immune cells	[59]
	Marrow	Observation of the behavior of bone marrow cells during bone marrow engraftment	[74]
Transgenic Mice	OT1	Observation of the spatial interaction between T cells and DCs	[75]
	OTI-CFP		
	OTI-DsRed		
	CD11c-YFP		
	CXCL10	Observation of the migration behavior of CD8^+^ T cells	[76]
	C57BL/6 Thy1.1		
	OT-I		
	Ds-Red		
	Hu-Mouse	Overcoming the differences in responses between model animal immune system and human immune system	[77]
	WAP-Myc	Observation of changes in the microenvironment in a model of breast tumor with spontaneous metastasis	[78]
	Cxcr6^+^/GFP	Analysis of the infiltration process of lymphocytes	[79]
	OT-I x GFP	Evaluation of changes in adoptive T cell morphology and migration in a solid tumor microenvironment	[80]
	DPE-GFP	Identification of a tumor-associated macrophage-mediated resistance pathway in anti-PD-1 therapy	[81]
	Cxcr6^+^/GFP	Long-term intravital imaging of a multicolor-coded tumor microenvironment	[82]
	Ccr2^−/−^ mice	Observation of the immunosuppressive behavior of Ly6Clo monocytes	[83]
	Cx3cr1/GFP		
	C57BL/6-Tg (CAG-EGFP) 1Osb/J	In vivo visualization of tumor antigen-containing microparticles	[84]
	Actb-EGFP C57BL/6	Observation of immunomodulatory and inhibitory functions of hepatic sinusoidal endothelial cells	[85]

## Abbreviations

ICIs	immune checkpoint inhibitors
IFN-γ	interferon-γ
CX3CL1/5/9/10/11	C-X3-C motif chemokine ligand 1/5/9/10/11
CXCR1/2	chemokine receptor cxcr1/2
NK cell	natural killer cell
DCs	dendritic cells
Tregs	regulatory T cells
MDSCs	myeloid-derived suppressor cells
CTLs	cytotoxic T lymphocytes
TGF-α/β	transforming growth factor-α/β
IL-1/2/10/12/15/24	interleukin-1/2/10/15/24
DcR3	decoy receptor 3
PD-L1	programmed death-ligand 1
ELISAs	enzyme-linked immunosorbent assays
WB	Western blotting
GFP	green fluorescent protein
LAG-3	lymphocyte-activation gene 3
CD19/27/40/137	memory T cell differentiation marker
GITR	glucocorticoid-induced TNFR-related gene
CEA-TCB	Cibisatamab
OX40	tumor necrosis factor receptor
CTLA-4	cytotoxic T lymphocyte-associated protein 4
VEGF	vascular endothelial growth factor
GM-CSF	granulocyte-macrophage colony-stimulating factor
CAR-T	chimeric antigen receptor-T
MHC	major histocompatibility complex
ICAM-1	intercellular adhesion molecule 1
TAA	tumor-associated antigen
NETs	neutrophil extracellular traps
TRM	tissue-resident memory
